# Supplementary material for: Addressing Vaccine Hesitancy in College Students Post COVID-19 Pandemic: A Systematic Review Using COVID-19 as a Case Study
Source: Vaccines (Basel). 2025 Apr 25;13(5):461. doi: 10.3390/vaccines13050461 (PMC12115507; doi:10.3390/vaccines13050461)
Supplement: Supplementary file 1 [file vaccines-13-00461-s001.zip › Supplementary Figure S1.pdf]

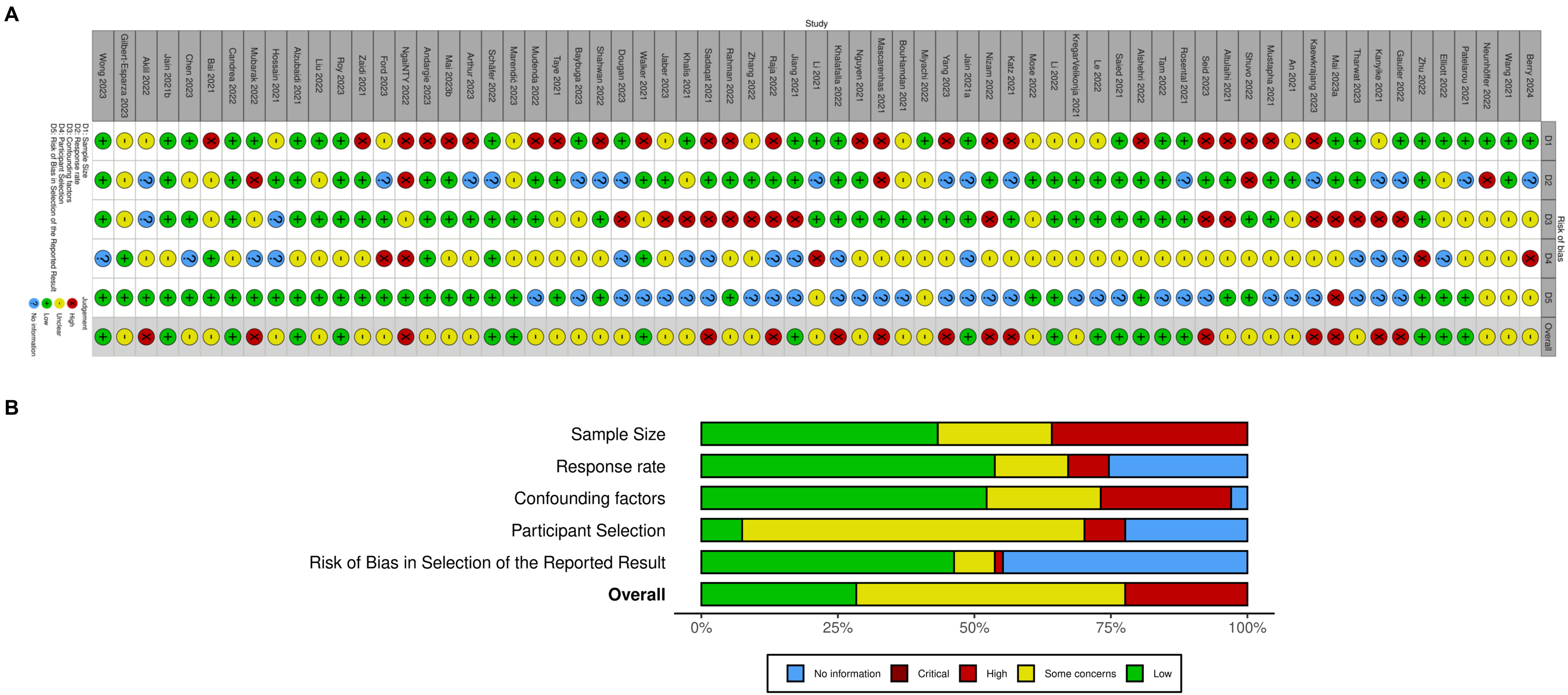

Supplementary Figure S1. Assessment of Risk of Bias. **A,** Traffic light plot summarizing the risk-of-bias assessment for each included study across five key domains: sample size (D1), response rate (D2), confounding factors (D3), participant selection (D4), and risk of bias in the selection of the reported result (D5). Each row represents an individual study, and each colored circle indicates the risk-of-bias level—green for “low,” yellow for “some concerns,” red for “high,” and dark red for “critical.” Gray circles indicate “no information.” This figure offers a detailed, study-specific visualization of methodological quality. **B,** Summary of risk-of-bias assessments for the included cross-sectional studies. Each horizontal bar represents the distribution of risk-of-bias ratings (no information, critical, high, some concerns, or low) across five key domains: sample size, response rate, confounding factors, participant selection, and risk of bias in selecting the reported result. The final bar shows the overall risk-of-bias rating across all domains. Color coding indicates the level of concern, with green representing “low,” yellow “some concerns,” red “high,” and dark red “critical.”
