# Supplementary material for: Addressing Vaccine Hesitancy in College Students Post COVID-19 Pandemic: A Systematic Review Using COVID-19 as a Case Study
Source: Vaccines (Basel). 2025 Apr 25;13(5):461. doi: 10.3390/vaccines13050461 (PMC12115507; doi:10.3390/vaccines13050461)
Supplement: Supplementary file 1 [file vaccines-13-00461-s001.zip › Supplementary Table S2.pdf]

Supplementary Table S2: Complete data extraction table for all included studies of our systematic review.

Note-taking			Study basics					Study design					Population inclusion criteria
No	Reviewer 1	Reviewer 2	Author	Year published	Manuscript title	Journal name	Objectives of the study	type of study	data collection methods	number of study sites included	data collection time	study setting (country)	Sample size
			Last name of first				copy the objective from the intro/abstract	RCT,cluster RCT,Pre-post,Quasi-experiment	Surveys, questionnaire		Time that the data was collected	country	n=
1	Willy	Xinyu	Berry	2024	Vaccine hesitancy and hesitant adoption among nursing students in Texas	Preventiv	The purpose of this study was to describe the level of vaccine hesitancy and hesitant adoption among nursing students in the state of Texas.	Descriptive study	Online suverys	1	Mid-April to mid	USA	599
2	Willy	Xinyu	Wang	2021	Factors influencing COVID-19 vaccination intention among overseas and domestic Chinese university students: a cross-sectional survey	Human V	This study aimed to explore the difference between overseas and domestic Chinese university	Cross-sectional study	Electronic questionn	1	January to Febru	China	833
4	Willy	Xinyu	Neunhöf	2022	Factors Associated with the COVID-19 Vaccination Status of Higher Education Students: Results of an Online Cross-Sectional Survey at Six	Vaccines	This cross-sectional study explored factors associated with the corona virus disease 2019	Cross-sectional study	Online surveys	1	July and Novem	Germany	6556
6	Willy	Xinyu	Patelarou	2021	Factors influencing nursing students' intention to accept COVID-19 vaccination: A pooled analysis of seven European countries.	Nurse Edu	To explore the intention of nursing students to get vaccinated for SARS-CoV-2	Cross-sectional study	Web surveys	7	December 2020-	Greece, Albania, C	2249
7	Willy	Xinyu	Elliott	2022	Vaccine acceptance and hesitancy among college students in Nevada: a state-wide cross-sectional study.	Vaccines	The purpose of this study was to identify factors related to COVID-19 vaccine acceptance	Cross-sectional study	Electronic surveys	1	February to Mar	USA	3773
8	Willy	Xinyu	Zhu	2022	Patterns and influencing factors of COVID-19 vaccination willingness among college students in China.	Vaccine	We aimed to examine the willingness to vaccination and influencing factors among	Cross-sectional study	Online surveys	30	March 18 to Apr	China	22,660
10	Willy	Xinyu	Gautier	2022	Cross-sectional study on COVID-19 vaccine hesitancy and determinants in healthcare students: interdisciplinary trainings on vaccination are needed.	BMC Me	The aim was to estimate vaccine hesitancy among healthcare students in France and to	Cross-sectional study	Online surveys	1	January 21 and F	France	1465
11	Willy	Xinyu	Kanyike	2021	Acceptance of the coronavirus disease-2019 vaccine among medical students in Uganda.	Trop Med	Unanimous uptake of the COVID-19 vaccine is required to subsequently avert its spread;	Cross-sectional study	Online surveys	10	Monday 15 Marc	Uganda	600
12	Willy	Xinyu	Tharwat	2023	Acceptance and hesitancy to receive COVID-19 vaccine among university students in Egypt: a nationwide survey.	Trop Med	The aim of this study was to identify acceptance and hesitancy to receive COVID-19	Cross-sectional study	Self-administered on	1	20th of July to 2	Egypt	1071
13	Willy	Xinyu	Mai	2023	A Cross-Sectional Research Study of COVID-19 Vaccine Acceptance Among Vietnamese Nursing Students	SAGE Op	The study aimed to assess the prevalence of vaccination acceptance and the factors	Cross-sectional study	Questionnaire	1	February to Mar	Vietnam	384
14	Willy	Xinyu	Kaewkra	2023	Anxiety, optimism, and COVID-19 vaccine hesitancy among students in a university in Southern Thailand during the 2021 academic year.	Vaccines (Basel)	This study examined the prevalence of COVID-19 vaccine hesitancy and its	Cross-sectional study	Online surveys	1	November and D	Thailand	409
15	Muxuan	Siran	Pham	2021	The intention to get a COVID-19 vaccine among the students of health science in Vietnam	Human Vaccines &	Specify the predictable factors of willingness to get a future COVID-19 vaccination	Cross-sectional study	Self-administered online questionnaire	1	NA	Vietnam	854
16	Muxuan	Siran	Mustaph	2021	Factors associated with acceptance of COVID-19 vaccine among university health sciences students in Northwest Nigeria.	PLoS ON	evaluate the factors associated with COVID-19 vaccine acceptance among health	Cross-sectional study	Online suverys	4	15 March 2021- 14 June 2021	Nigeria (Northwest	440
17	Muxuan	Siran	Shuvo	2022	Factors associated with intention to take COVID-19 vaccine among the university students in Bangladesh.	Clinical and Experime	Evaluate the intention to take COVID-19 vaccines among the Bangladeshi university students	Self-administered question survey	Online surveys	1	August 7 to September 18, 2021	Bangladesh	310
18	Muxuan	Siran	Altulaihi	2021	Factors and Determinants for Uptake of COVID-19 Vaccine in a Medical University in Riyadh, Saudi Arabia	Cureus	Explore the perception and knowledge of medical students ar KSAU-HS towards COVID-	Cross-sectional study	web-based survey	NA	NA	Riyadh, Saudi Arab	204
19	Muxuan	Siran	Seid	2023	COVID-19 vaccine acceptance, use, and associated factors among health sciences students in Eastern Ethiopia	Journal of Public Health	Aim to access COVID-19 vaccine acceptance, use, and associated factors among health	Cross-sectional study	structured questionai	NA	August 10-30, 2022	Eastern Ethiopia	403
20	Muxuan	Siran	Rosental	2021	Integrating health behavior theories to predict COVID-19 vaccine acceptance: differences between medical students and nursing	Vaccines	This research aimed to explore behavioral-related factors based on both the Health Belief Model	Cross-sectional online study	Questionnaire	1	27 August to 28 September 2020	Israel	628
21	Muxuan	Siran	Tam	2021	Factors associated with decision making on COVID-19 vaccine acceptance among college students in South Carolina.	Psychology, Health &	Explore COVID-19 vaccine acceptance among college students in South Carolina and	Cross-sectional study	Online surveys	NA	September to October 2020	South Carolina	1062
22	Muxuan	Siran	Alshehri	2022	Knowledge and attitudes towards COVID-19 vaccination among medical and paramedical students	Medical science	assess the knowledge and attitude towards the COVID-19 vaccine among medical and	cross-sectional, survey-based study	online questionaire	1	NA	Saudi Arabia	465
23	Muxuan	Siran	Saied	2021	Vaccine hesitancy: beliefs and barriers associated with COVID-19 vaccination among Egyptian medical students.	Journal of Medical	The study aimed to explore the level of COVID-19 vaccine hesitancy and determine the	Cross-sectional study	online questionaire	2	During Jan 2021	Egypt	2133
24	Muxuan	Siran	Le	2022	Predictors of COVID-19 vaccine acceptability among health professions students in Vietnam.	BMC Public Health	This study applied the Health Belief Model to determine predictors of COVID-19	Cross-sectional study	Online surveys	NA	1st April to 30th June 2021	Vietnam	911
25	Muxuan	Siran	Velikonj	2021	Attitudes of Nursing Students towards Vaccination and Other Preventive Measures for Limitation of COVID-19 Pandemic: Cross-Sectional Study in	Healthcare	The aim of this study was to investigate adherence to preventive measures and	Cross-sectional study	web-based survey	NA	2 February–5 March 2021	Three European: Slovenia, Poland, Sebria.	872
26	Muxuan	Siran	Li	2022	Chinese University Students' Awareness and Acceptance of the COVID-19 Vaccine: A Cross-Sectional Study	Risk Management and	This study aimed to assess the awareness and acceptance of the COVID-19 vaccine among	Cross-sectional study	anonymous survey via face-to-face interview and the	NA	10 to 28 June 2021	China	721
27	Muxuan	Siran	Mose	2022	COVID-19 vaccine hesitancy among medical and health science students attending Wolkite University in Ethiopia.	PLoS ON	this study aimed to assess COVID-19 vaccine hesitancy and its associated factors among	Cross-sectional study	Self-administered and structured questionnaire	1	March 1 to 30, 2021	Ethiopia	420
28	Muxuan	Siran	Katz	2021	COVID-19 Vaccination Compliance and Associated Factors among Medical Students during an Early Phase of Vaccination Rollout-A	Vaccines	This population-representative survey in Israel aimed to investigate whether the	population-representative survey	anonymous questionaire	1	Dec-20	Israel	104
30	Xinyu	Willy	Nizam	2022	Analyzing COVID-19 Vaccine Hesitancy among University Students in UAE: A Cross-Sectional Study	Dubai Me	This study aims to explore the att	Cross-sectional study	Questionnaire	1	April-June, 2021	UAE	385
31	Xinyu	Willy	Jain	2021	COVID-19 vaccine hesitancy among medical students in India.	Epidemio	The present study aims to assess	Cross-sectional study	Questionnaire	1	2 February-7 Ma	India	1068
32	Xinyu	Willy	Yang	2023	Attitude and influencing factors to receive the COVID-19 vaccine among university students in Sichuan Province, China: A cross-sectional	Nursing C	To explore the university studen	Cross-sectional study	Questionnaire	1	June, 2021	China	397
34	Xinyu	Willy	Miyachi	2022	COVID-19 Vaccine Intention and Knowledge, Literacy, and Health Beliefs among Japanese University Students.	Vaccines	To reveal the COVID-19 vaccin	Cross-sectional study	Questionnaire	1	March-May, 202	Japan	1776
35	Xinyu	Willy	BouHam	2021	COVID-19 vaccine hesitancy among university students in Lebanon.	Epidemio	The purpose of this study was to identify the readiness, behavioural intentions and	Cross-sectional study	Online surveys	1	11 May 2021-18	Lebanon	800
36	Xinyu	Willy	Mascare	2021	Dental students' attitudes and hesitancy toward COVID-19 vaccine	The voice	The purpose of this study is to assess the attitudes of dental students to the novel	Cross-sectional study	Online surveys	3	2020	USA	248
37	Xinyu	Willy	Nguyen	2021	Predictors of Intention to Get a COVID-19 Vaccine of Health Science Students: A Cross-Sectional Study	Risk Man	The aim of this study is to identi	Cross-sectional study	Questionnaire	1	April, 2021	Vietnam	412
40	Xinyu	Willy	Khalafal	2022	COVID-19 Vaccine Hesitancy Prevalence and Predictors among the Students of Jazan University, Saudi Arabia Using the Health Belief Model:	Vaccines	This study aims to determine the	Cross-sectional study	Questionnaire	1	March-August, 2	Saudi Arabia	1039
42	Xinyu	Willy	Li	2021	Hesitancy toward COVID-19 vaccines among medical students in Southwest China: a cross-sectional study.	Human V	This study aims to inform the va	Cross-sectional study	Questionnaire	1	March 15-March	China	2196
44	Siran	Muxuan	Jiang	2021	Nursing students' attitudes, knowledge and willingness of to receive the coronavirus disease vaccine: A cross-sectional study	Nurse Educ Pract	to investigate the knowledge, attitudes and willingness of nursing students to receive the	Cross-sectional study	Questionnaire	1	from February to April 2021	China	1488
49	Siran	Muxuan	Raja	2022	COVID-19 vaccine acceptance, hesitancy, and associated factors among medical students in Sudan.	PLoS One	to determine the acceptance and hesitancy of the COVID-19 vaccine and associated factors	cross-sectional study	Questionnaire		from 30th June to 11th July 2021.	Sudan	217
50	Siran	Muxuan	Zhang	2022	Determinants of COVID-19 Vaccine Acceptance and Hesitancy: A Health Care Student-Based Online Survey in Northwest China	Front Public Health	to collect information regarding their attitudes, beliefs, and acceptance of COVID-19	cross-sectional study	questionnaire		from August 16–20, 2021,	China (Gansu)	631
51	Siran	Muxuan	Rahman	2022	Knowledge, attitude, and hesitancy towards COVID-19 vaccine among university students of Bangladesh.	PLOS ONE	to examine the knowledge, attitude, and hesitancy towards the vaccine	cross-sectional study	Questionnaire		March 12 to April 02, 2020	Bangladesh	449
52	Siran	Muxuan	Sadaqat	2021	Determination of COVID-19 Vaccine Hesitancy Among University Students	Cureus	to determine the association between university curricula and the degree of hesitancy for the	cross-sectional study	questionnaire		6 months	Pakistan	415
53	Siran	Muxuan	Khalis	2021	COVID-19 Vaccination Acceptance among Health Science Students in Morocco: A Cross-Sectional Study.	Vaccines	to determine the willingness and identify predictive attitudes and beliefs of COVID-19 vaccine	Cross-sectional study	Questionnaire		in Jan. 2021	Moroco (Casablanca)	1272
54	Siran	Muxuan	Jaber	2023	COVID-19 Vaccine Acceptance among Ajman Undergraduate Dental Students: A Cross-Sectional Study.	Int J Dent	to assess the knowledge, attitudes, and behaviors of undergraduate dental students	Cross-sectional study	Questionnaire		in Jan. 2022	UAE	624
55	Siran	Muxuan	Walker	2021	Vaccine Acceptance and Its Influencing Factors: An Online Cross-Sectional Study among International College Students Studying in China.	Vaccines (Basel)	to explore the factors that influence the acceptance of the COVID-19 vaccine among	Cross-sectional study	Questionnaire		from 17 March to 26 March 2021	China (Jiangsu)	330
56	Siran	Muxuan	Dougan	2023	Factors associated with COVID-19 vaccination status: A cross-sectional study of California students in higher education.	Preventive Medicine	To examine the factors associated with coronavirus vaccine hesitancy among	Cross-sectional study	Questionnaire		between June and August 2021	U. S. (Carlifornia)	4444
57	Kangpei	Zixuan	Shahwan	2022	Prevalence, Knowledge and Potential Determinants of COVID-19 Vaccine Acceptability Among University Students in the United Arab Emirates:	Journal of Multidisci	To evaluate the prevalence, knowledge, attitude and acceptance of the COVID-19	Cross-sectional study	Questionnaire	1	from 6 March to 20 August 2021	UAE	467
59	Kangpei	Zixuan	Baybuga	2022	COVID-19 vaccination intent in university students and influencing factors: An analytical cross-sectional study	Journal of the Pakistan	To determine coronavirus disease-2019 vaccination intent and factors influencing the	Cross-sectional study	Questionnaire	1	from 25 January - 25 February 2021	Turkey (Mugla)	1069
60	Kangpei	Zixuan	Taye	2021	Coronavirus disease 2019 vaccine acceptance and perceived barriers among university students in northeast Ethiopia: A cross-sectional	Clinical Epidemiology and	This study aimed to assess vaccine acceptance, associated factors, and perceived barriers	cross-sectional study	questionnaire	1	from 20 to January 30, 2021	Northeast Ethiopia	423
61	Kangpei	Zixuan	Mudenda	2022	Awareness and acceptance of COVID-19 vaccines and associated factors among pharmacy students in Zambia.	Malawi Medical Journal	This study aimed to assess the awareness and acceptance of COVID-19 vaccines and	Cross-sectional study	questionnaire	1	from February to April 2021	Lusaka, Zambia	326
62	Kangpei	Zixuan	Marendi	2023	Determinants of COVID Vaccination Willingness among Health and Non-Health Studies Students: A Cross-Sectional Study.	vaccines	The aim of this study was to evaluate the overall attitudes towards COVID vaccination	Cross-sectional study	questionnaire	1	This cross-sectional study was conducted	Croatia	752
63	Kangpei	Zixuan	Schafer	2022	Determinants of university students' COVID-19 vaccination intentions and behavior.	scientific reports	Using a German university as an example and based on an online survey of 1398 university	Cross-sectional study	online surveys	1	between June 21 and August 15, 2021	German	1114
64	Kangpei	Zixuan	Arthur	2023	Acceptability and perception of COVID-19 vaccines among foreign medical students in China: A cross-sectional study.	frontiers in Public Health	This study, therefore, fills the literature gap using a focus group technique to	Cross-sectional study	Online surveys	1	from May 2021 to April 2022.	China	403
65	Kangpei	Zixuan	Mai	2023	COVID-19 Vaccine Acceptance Among Healthcare Students in Vietnam, Based on Health Belief Model	SAGE Open Nursing	The study aimed to assess the prevalence of vaccination acceptance and the factors influencing COVID-19 vac cine acceptance among Vietnamese healthcare	Cross-sectional study	Questionnaires	1	from February to March 2022.	Vietnam	384
66	Kangpei	Zixuan	Andargie	2023	Covid-19 vaccine uptake and associated factors among health science University students in northeastern Ethiopia, a cross-	Human Vaccines &	The aim of this study is to assess COVID-19 vaccine uptake and associated factors among health	Cross-sectional study	Questionnaires	1	from 1 to 15 July, 2022	Northeast Ethiopia	403
67	Kangpei	Zixuan	Ngai	2022	Evaluating the attitudes and behavior of Hong Kong medical students toward receiving the COVID-19 vaccine.	Human Vaccines &	. This study primarily aimed to determine the population of medical students	Cross-sectional study	questionnaire survey	1	from 16 September 2021 to	Hong Kong, China	214
68	Kangpei	Zixuan	Ford	2023	Factors influencing Australian nursing and midwifery students COVID-19 vaccination intentions.	Vaccine:	The aim of this study was to explore factors influencing Australian nursing and	Cross-sectional mixed method study	online survey platfo	1	between January 2022 and May 2022	Australia	556
69	Kangpei	Zixuan	Zaidi	2021	Attitudes and intentions toward COVID-19 vaccination among health professions students and faculty in Qatar.	vaccines	This cross-sectional study examines the impact of psychological and	cross-sectional study	online survey	1	(from 3 March 2021 to 31 March 2021	Qatar	364
70	Kangpei	Zixuan	Roy	2023	Multi-dimensional potential factors influencing COVID-19 vaccine booster acceptance and hesitancy among university academic	PLoS One	This study investigates COVID-19 vaccine booster dose acceptance and com pares the	cross-sectional study	questionnaire	51 public and 108 pr	between 15th June, 2022 and 15th August,	Bangladesh	745
72	Zixuan	Kangpei	Liu et al	2022	Willingness and Influencing Factors to Receive COVID-19 Vaccination Among Chinese Medical Students	Frontiers	The aim of this study is to evaluate the desire of medical students in China	Cross-sectional study	Online surveys	NA	between 1 Marc	China	3047
73	Zixuan	Kangpei	Alzubaid	2021	A mixed-methods study to assess COVID-19 vaccination acceptability among university students in the United Arab Emirates.	HUMAN	investigate university students' attitudes in the United Arab Emirates (UAE)	Mixed-method study	Online surveys & int	1	between 16th-24	Sharjah	669
74	Zixuan	Kangpei	Hossain	2021	COVID-19 vaccine acceptability among public university students in Bangladesh: Highlighting knowledge, perceptions, and attitude	HUMAN	assess the COVID-19 vaccine-related knowledge, perceptions,	Cross-sectional study	Online surveys	NA	from March 22,	Bangladesh	900
76	Zixuan	Kangpei	Mubarak	2022	Beliefs, barriers, and acceptance associated with COVID-19 vaccination among Taif University students in Saudi Arabia.	Journal of	assess the beliefs, barriers, and a	Cross-sectional study	Online surveys	1	from March 202	Saudi Arabia	332
77	Zixuan	Kangpei	Candrea	2022	COVID-19 VACCINE ACCEPTANCE AMONG MEDICAL STUDENTS IN ROMANIA	Journal of	focus on a homogeneous group	Cross-sectional study	Online surveys	1	NA	Finland	725
79	Zixuan	Kangpei	Bai et al	2021	Attitudes toward COVID-19 vaccines in Chinese college students.	Internatio	examined the attitudes of Chinese college students toward COVID-19	Cross-sectional study	Online surveys	1	from December 27, 2020 to January 18,	China	2892
80	Zixuan	Kangpei	Chen et a	2023	Barriers and facilitators to COVID-19 vaccine uptake among Australian health professional students during the pandemic: a nationwide study.	Journal of	Using a cross-sectional online survey we investigated knowledge, attitudes, and risk	Cross-sectional study	Online surveys	1	from October 20	Australia	1114
81	Zixuan	Kangpei	Jain	2021	Factors Influencing COVID-19 Vaccination Intentions Among College Students: A Cross-Sectional Study in India.	Front Pub	Understanding their knowledge about coronavirus disease 2019 (COVID-19),	Cross-sectional study	Online surveys	1	between November 2020 and January	India	655
82	Zixuan	Kangpei	Aklil	2022	Willingness to Receive COVID-19 Vaccine and Associated Factors Among College Students in Gondar City, Northwest Ethiopia	Frontiers	aimed to assess willingness to re	Cross-sectional study	Online surveys	1	from January 12	Ethiopia	384
83	Zixuan	Kangpei	Gilbert-E	2023	Vaccine hesitancy in college students.	Vaccines	investigate COVID-19 vaccine hesitancy in	Cross-sectional study	Online surveys	1	June and July 2	USA	311
85	Zixuan	Kangpei	Wong et	2022	Factors influencing COVID-19 vaccination intention among university students: A cross-sectional study in Malaysia.	Biosafety	measure the COVID-19 knowle	Cross-sectional study	Online surveys	1	21-Jul	Malaysia	1274

Note-taking									Vaccination status		Vaccine hesitancy				Vaccine Influencing factors						WHO 3C model Complacancy (Perception of risk)	
No	Population characteristic	Gender info			Ethnicity		range of age	Health status	Vaccination doses	number (%)	Intention to receive COVID 19 vaccine				Influencing factors 1	Number of patients (%)	Influencing factros 2	Number of patients (%)	Influencing factors 3	Number of patients (%)	Susceptibility	
	for example (nursing student, general college student)	number of male students	number of female students	male : female ratio	highest ethnicity of the patients	n (%)	18+/40+	Healthy or with what disease	1 dose/ 2 dose/ all doses		Yes (n=)	percentage	No (n=)	percentage		if mentioned in the articles, include the		if mentioned in the articles, include the		if mentioned in the articles, include the	Individual	Community
1	Nursing student	69	525	1：7.6	White or Caucasian	339 (56.6%)	18+	NA	1 dose	84.80%	507	85%	92	15.40%	Side effects	341 (56.9%)	Personal freedon	249 (41.6%)	Trust issues (Dis	83 (13.9%)	Majority of respondents (85%) received at least one dose of the COVID-19 vaccine.	27% of respondents cited protecting others in the community as a reason for getting vaccinated.
2	Overseas and Chinese Univers	314	519	1：1.65	Chinese	833 (100%)	18-30	NA	-	-	332	65.40%	176	34.60%	Perceived Barrie	233 (28%)	Perceived benef	308 (37%)	Cues to Action	292 (35%)	29.2% of overseas students and 22.2% of domestic students	54.6% of overseas students and 55.5% of domestic students are
4	Univeristy student in southwe	2226	4106	1 ： 1:85	German	95.20%	18-70 years	9.4% had pr	All doses	2882 (43.9%)	2882	43.90%	3674	56.10%	Safety concerns	3674 (56.1%)	Lack of Trust in	2458 (37.5%)	Influence of Soc	1965 (30.0%)	28% of students believed they were at risk of contracting	35% of students were concerned about spreading COVID-19
6	nursing student	344	1902	1: 5.54	Kosovo	1020 (45.4%)	18+	Majority wit	NA	NA	985	43.80%	1264	56.20%	Doubts about th	1129 (72.4%)	Belief that even	99 (6.3%)	Belief that the v	84 (5.4%)	Majority of students were aware of the risk of COVID-19, with	Higher proportions of students from Italy (71.2%) and Spain
7	Undergraduate and graduate st	1012	2546	1012 : 2546	White	54.90%	18-30	NA	All doses	714 (18.9%)	2037	54%	888	23.50%	Side effects	598 (67.3%)	Belief that more	660 (74.3%)	Suspicion of vac	222 (25%)	Nearly one quarter (23.5%) of respondents expressed	27.3% of respondents indicated a desire to protect others in the
8	General college students	9146	13514	1：1.47	Han ethnicity	92.80%	15-31	13.0% repor	NA	NA	13,738	60.60%	1,363	6%	Side effects	11,662 (58.3%)	Family members	7,798 (28.6%)	Actively searchi	19,440 (82.3%)	A significant proportio	A smaller proportion (6
10	Healthcare student	246	1219	1 ： 4.95	NA	NA	18+	NA	NA	NA	862	55.50%	703	44.50%	Side effects	319 (54.7%)	Prefer to wait fo	456 (76.2%)	Prefer preventiv	196 (35.1%)	15% of healthcare students reported having been infected	NA
11	Medical student	377	223	1.69:1	NA	NA	≤ 24 years	NA			224	37.30%	376	62.70%	Side effects	242 (64.4%)	Negative inform	201 (53.5%)	Doubts about va	136 (36.2%)	Among the participants, 188 (30.5%) perceived a	225 (37.5%) of students think that COVID-19 poses a
12	University students (both med	341	730	1 ： 2.14	NA	NA	Mean age 20	NA	NA	NA	739	69%	332	31%	Side effects	829 (77.4)	Doubts about va	201 (60.5% of V	Preference to wa	NA	48.6% thought they were not at high risk to get COVID-19.	NA
13	Healthcare students from various fields including medicine, nurse and midwife,	68	316	1： 4.65	NA	NA	18+	Majority per	NA	NA	352	91.70%	32	8.30%	Perceived Good	252 (65.6%)	COVID-19 Infec	202 (52.6%)	NA	NA	NA	High perception of risk
14	University students from Prince of Songkla University	91	318	1 ： 3.5	NA	NA	18+	Mostly healt	2 doses	88.30%	400	97.80%	9	2.20%	Safty of vaccine	8 out of 9 hesitant students (88.9%)	Trust in Public Authorities	7 out of 9 hesitant students (77.8%)	Side effects	7 out of 9 hesitant students (77.8%)	18.8% perceived themselves as at risk of COVID-19 infection.	NA
15	first to sixth-year health sciences students who were studying Medicine, Preventive	316	538	1 ： 1.7	NA	NA	22.1±2.5	NA	NA	NA	657	77.10%	196	22.90%	Side effects	143 out of 196 hesitant students (73%)	A desire to delay and follow up on the	128 out of 196 hesitant students (65.3%)	Afraid of the process of new vaccine	105 out of 196 hesitant students (53.6%)	27.6% afraid of needles, 43.9% have a plan to use other	18.4% not consider themselves in the high-risk group
16	health sciences students (including Medicine, Pharmacy, Nursing, and	216	224	1 ： 1.03	Hausa	281(63.9%)	18+	NA	NA	NA	176	40%	264	60%	Previously refused other vaccines	NA	Hausa tribe was found to be less likely to accept	NA	NA	NA	A low perceived risk of infection among medical students was	NA
17	university students of Banglad	156	154	1 ： 1	NA	NA	18-25	6.1% poor, 49% good, 44.8% fair	NA	NA	285	91.90%	9	2.90%	COVID-19 vaccines are not safe	81.60%	Side effect	62.60%	the effectiveness of COVID-19 vaccine is very	50%	Approximately 70% are worried of getting COVID-19 themselves	Over 75% think there could be a new outbreak of COVID-
18	medical students in their clinical years at the College of Medicine in King Saud bin	118	86	1.37 ： 1	NA	NA	19-29	NA	NA	NA	135	66.20%	69	33.80%	Fear of unknown adverse effects	approximately 21%	Fear of long-term genetic effects (which	15%	Doubts about vaccine efficacy	approximately 14%	Fear of being infected with COVID-19 (approximately 19%)	Fear of infecting the family with COVID-19, especially parents
19	The study population was all students of health and medicine sciences from	259	144	1.79 ： 1	Muslim	40.70%	not less than 19; mean age 23.62	NA	1 dose: 69 (17.1%)/2 doses: 49 (12.2%)/ More dose: 8 (2%)		203	50.40%	200	49.60%	Academic third year	52 (71.2%) (N=	History of family	31 (50.8%) (n=6	NA	NA	Fear of being infected with COVID-19 (19.1%)	Fear of infecting the family with COVID-19(3.2%)
20	Nursing students and medical students	210	418	1 ： 2	Jewish	90.20%	18+	16.9% with chronic diseases	NA	NA	652	78.34%	180	21.66%	Safety and quality of the vaccine	NA	Not tried on others	NA	Low efficacy	NA	"Natural immunity is preferable"	NA
21	full-time students currently enrolled in a university in South Carolina	211	848	1 ： 4	White/Caucasian	912 (85.9%)	18+	NA	NA	NA	644	60.60%	258	24.30%	Side effects	NA	Ways the vaccines will be administered	NA	Where the vaccines were made (domestic	NA	NA	NA
22	paramedical and medical students from Al-Rayan colleges	228	237	49 ：51	Arabian	60%	18-29	Very good 17.6%, good	NA	NA	124	26.70%	97	20.90%	The preparation of the Corona vaccine was	245 (52.7%)	Doubts about vaccine efficacy	235 (50.5%)	low likelihood of complications	230 (49.5%)	49.5 % students think they have a low likelihood of	Low concern
23	medical students in Tanta and Kafrelsheikh Universities	742	1391	1 ： 1.9	NA	NA	average 20.2	Very good 17.4%, good	NA	NA	746	34.90%	413	19.40%	Insufficient information regarding the	1582 (74.17%)	Insufficient information regarding the	1552 (72.76%)	The financial cost hindrance if the vaccine is	1451 (68%)	Fear of being infected with COVID-19 (35.11%)	Fear of infecting the family with COVID-19, especially parents
24	health professions domestic and foreign students in Dong Thap province, Vietnam	224	687	1 ：3	NA	NA	average 20.7	Very good 20.9%, good	NA	NA	525	58%	14	1.50%	Adverse effects of vaccine cause death	359 (39.5%)	Difficult access to vaccination if not free	366 (40.3%)	vaccine not safe due to rapid research	263 (28.9%)	Unvaccinated students infected with SARS-COV-2 during hospital	NA
25	nursing students of the 1st and the 2nd level of education	775	97	1 ： 9	NA	NA	average 23.5(6.5)	NA	at least 1 dose	213(24%)	305	35%	192	22%	Fear of side effects	NA	General vaccination refusal	NA	Time	NA	Perceived high risk if contracting illness	Protection of parents
26	Respondents enrolled in Chinese universities, 18 years old or older, and fluent in	224	497	1 ： 2.2	Han group	685 (95%)	18-23		NA	NA	630	87.40%	18	2.50%	They thought themselves at low risk of	55.60%	Fear of side effects	44.40%	They can protect themselves in	44.40%	90 % participants believe vaccine is good for oneself and low	59.7% of them believe vaccine is beneficial to others and society
27	medical and health science students attending Wolkite University	249	272	1 ：0.69	NA	NA	22.7±1.5	NA	NA	NA	247	58.80%	173	41.20%	Fear of side effects	39%	Concerned about its safety	24%	concerned about its efficacy	20%	Fear of being infected with COVID-19 (49.7%)	Fear of infecting the family with COVID-19 (36.8%)
28	medical students at the Azrieli Faculty of Medicine of Bar-Ilan University, Safed, Israe	40	64	1 ： 1.6	NA	NA	23-29	Health 93 (89.4%), with health	f the students were vaccinated		95	91.30%	9	8.70%	lack of knowledge about the	NA	exposure to COVID-19 vaccine-related	NA	NA	NA		
30	University students in UAE	90	295	1：3.28	not specifically menti	NA	16-35	Out of 385 p	NA	NA	257	66.75%	128	33.25%	Side effects	226 (58.7%)	Concerns over th	116 (30.1%)	Advised against	61 (15.8%)	In terms of vaccination rates, 257 (66.75%) students have	NA
31	Medical students in India	549	519	1：0.945	not specifically menti	NA	not specifica	NA	NA	NA	955	89.40%	113	10.60%	concern over saf	82.30%	concern over eff	28.30%	perceiving lowe	14.20%	Among those who agreed the statement "I am willing to take	Overall, more than three-fourths medical students viewed that
32	university students in Sichuan	117	280	1：2.39	Han Chinese	366 (92.2%)	18+	20 (5%) part	NA	NA	339	85.40%	58	14.60%	concern about th	44.80%	lack of informati	31%	concern about th	29.30%	The scoring rate of peo	NA
34	Japanese university students	951	824	1：0.866	not specifically menti	NA	20.9±4.1	not mention	NA	NA	1007	56.70%	156	8.80%	perceived severi	Crude odds ratio	Perceived benefi	Crude odds ratio	perceived barrie	Crude odds ratio	The odds ratio for "I m	The odds ratio for " I m
35	university students in Lebanon	325	433	1：1.33	Lebanese	646 (85%)	18+	377 (50%) p	NA	NA	720	90%	80	10%	No sense to get	39 (5%)	Personal freedon	43 (6%)	Government is u	58 (8%)	136 participants believe	NA
36	dental students at three geogra	104	144	1：1.38	White	138 (55.5%)	26.3±3.8	10.6% of res	NA	NA	138	55.80%	110	44.20%	Be likely to be e	94.70%	Concern over ef	54.10%	Concern about s	43.50%	94.7% respondents agr	NA
37	health science students in Tra	189	223	1：1.18	not specifically menti	NA	22.5±2.8	NA	NA	NA	320	77.70%	92	22.30%	Safety concerns	67.40%	development iss	58.70%	Side effects	79.30%	Overall score of percei	NA
40	Students of Jazan University	419	620	1：1.48	not specifically menti	NA	15-30	not mention	NA	NA	869	83.60%	170	16.40%	Perceived suscep	86.50%	perceived benefi	69%	perceived barrie	42.20%	38.6% of respondents b	23.4% of respondents a
42	medical students in a medical	402	1794	1：4.46	not specifically menti	NA	17.66±2.25	not mention	NA	NA	1292	58.80%	904	41.20%	Concern over ef	13.60%	Concern over sic	32.70%	the risk of gettin	7.74%	7.74% of respondents a	44.8% of respondents a
44	nursing college students	234	1254	1: 5.36	Han	98.52%	23-				1240	84.38%	248	16.67%	worry about infecting others	scoring rate 88.8	significant impact of COVID-19 on	scoring rate 77.6	careness about v	scoring rate 76.	participants believe contracting COVID-19 has a significant impact	participants believe it will affect the people around you or the
49	medical students	100	117	1: 1.17	non- Sudanese	0.714	161 (74.2%) not over 24; 56		not mentioned		121	55.80%	25	11.50%	history of COVID-19 infection	62 (28.5%)	belief that vaccines are generally safe	152 (70.04%)	confidence that the vaccine can end the	122 (56.22%)	13.5% participants think they are young, and they can recover	NA
50	health care students	128	503	1: 3.93			61.17% aged below 22	Healthy 622 (98.57%); other			491	77.80%	71	11.30%	believed in the effectiveness of the COVID-19	569 (90.17%)	felt it is their responsibility to receive the	526 (83.36%)	thought COVID-19 to be very severe	479 (75.91%)	24.09% participants believe COVID-19 is only generally seriou	NA
51	general college students	232	217	1: 0.935			not mentioned		not mentioned		332	73.94%	117	26.10%	Fear of side effects	102 (87.18%).	lack of information	83 (70.94%)	Other reasons (including ineligibility and	53 (45.30%)	NA	85.75% of participants were concerned about the pandemic.
52	219 (52.8%) medical students; 196 (47.2%) non-medical students.	133 (32%)	282	1 :2.12			18-25				407	98.07%	8	1.93%	no known one had side effects	264 (63.6%)	vaccine is tested	252 (60.6%)	no ond het sick from vaccine	216 (52.0%)	30% participants claim that known person died from COVID-19	41.2% participants believe vaccine can protect vulnerable in
53	general college students	402 (31.6%)	870	1: 2.16			652 (51.3%) no older than	1104 (86.8%) no chronic			343	26.90%	929	73.10%	knowledege of possible side effects	1064 (83.6%)	durantion of protection	540 (42.5%)	Health status	538 (42.3%)	NA	NA
54	undergraduate dental surgery students	307	307				18-26				144	23.10%	50	8%	Want to know more about how it works	420 (67.3%)	The vaccine was effective about 60%of	304 (48.7%)	Many people experienced minor side	262 (42.0%)	10.6% participants refuse vaccine because they do not think they	NA
55	international college students	180 (54.5%)	150	1: 0.83	African	165 (50%)	18+	very good/good 330			120	36.40%	101	30.60%	believe vaccines prevents diseases	228 (69.1%)	worry that people could have been	227 (68.8%)	believe vaccination reduces their	221 (66.9%)	48.2% participants believe there is a possibility of being	42.8% participants are concerned about those around them being
56	general college students in Carlifornia	1120 (25.2%)	2269	1 :2.03	White	0.269	15-83		NA	NA	3631	82%	813	18%	Side effects	61 (7.5%)	developed too fast	49 (6.02%)	safety	43 (5.28%)	NA	NA
57	the students, both UAE nationals and non-national residents, at Ajman University	109(23.4%)	358	109 : 358	Arabic	383 (82%)	most of them were aged below		NA	NA	263	56.30%	204	43.70%	unforeseen impacts	306 (65.5%)	Pharmaceutical companies are going to	288 (61.7%)	mistrust of vaccines benefits	164 (35.1%)	More than half participants wrongly identified that nausea,	NA
59	undergraduate students enrolled in various departments at the	440 (41.2%)	629	1：1.43	NA	NA	The mean age of the sample was	NA	NA	NA	578	54.10%	169	15.80%	Vaccines weredevelopedin	285(26.7)	Immediatesideef	181(16.9)	Theexact reason whyvaccines weredevelopedis	145(13.6)	48.4 percent belive to be at risk of COVID-19	NA
60	All graduating class students of Debre Berhan University were the source population,	281 (66.4)	142	1：0.5	NA	NA	<25: n=245 >=25: n=178	NA	NA	NA	293	69.30%	130	30.70%	had a need to know more about the	48%	Uncertainty of vaccine effectiveness	52%	had concern on side effects	68%	The main reasons (perceived barriers) for participants'	NA
61	undergraduate pharmacy students enrolled in the pharmacy degree programme	164 (50.3%)	162	1：0.99	Religion: Christianity	322 (98.8%)	18-23 (48.2%) 23+	NA	NA	NA	80	24.50%	246	75.50%	Religion	The current study found that religion was	Safety	193 (78.5)	Need	28 (11.4)	NA	NA
62	This study involved students from the University of Split's four faculty institutions: the	112 (15%)	640	1：0.175		NA	Age median: 21	Presence of chronic Diseases:	NA	NA	206	27.40%	286	38%	Fields of study	the results show that a slight majority	Opinions about epidemiological measures	The majority (51.7%) of students who	Source credibilit	. Furthermore, the majority of students who	NA	NA
63	all students of the university were invited to a cross-sectional online health	270	844	1：3.02	NA	NA	M=23.5	NA	at least one dose	69.90%	778	69.90%	336	30.10%	Confidence	NA	Constrains	NA	Field of study	NA	Association of Complacancy with the likelihood of	NA
64	all the medical students studying medical-related majors in	246 (63.7)	140	1：0.57	NA	NA	20-40: 353 (91.5)	NA	NA	NA	322	83.42%	36	9.33%	Gender	NA	How many doses do you think you can	NA	had concerns about the vaccine's safety	37.30%	NA	NA
65	The correspondents were recruited from 3,203 university	312	72	1：4.348	NA	NA	mean age: 20.51+-1.55 years	The majority of respondents	NA	NA	352	91.57%	32	8.43%	Fields of study	Med: 87% Yes, Phar: 87%, Nurse: 96%,	Perceived health status	Excellent: 95% Yes, Good: 92.0%, Not	COVID-19 infection among relatives or	Yes: 95% yes, No: 87% yes	Perceived susceptibility: Yes 11.39+-1.79 (Health	NA
66	health science students at Wollo University	273	130	1：0.476	NA	NA	20-21：39%, 22-	Having Known Disease:	Uptake of COVID-19 vaccine – defined as the number of	NA	94	23.30%	309	76.70%	If not vaccinated, transmit to	44 10.9	Best prevention method	56 13.9	Fear of side effe	68 16.9	NA	NA
67	Current medical students from The University of Hong Kong (HKU) were	111	103	1：0.93	NA	NA	Clinical and Preclinical students.	NA	Received 2 doses Received 1 dose None (0 dose)	2: 210 98.1%/1: 3 1.4%/ 0: 1 0.5%	213	99.50%	1	0.50%	Fear of potential serious side effects	45 61.6%	Waiting to assess outcomes of other people	40 (54.8%)	Busy work/study schedule	29 (39.7%)	NA	NA
68	Participants were eligible if they were aged 18 years or older and were currently	30	548	1：18.27	NA	NA	18-24:198(34)	25-34 193(33.1)	NA	NA	512	95%	27	5%	Protect Myself	363(71)	Protect the Community	342(66.9)	To Enable Employment	281(55)	My Chance of getting COVID-19 in the next few months is high:	
69	health professions students and faculty of different nationalities at Qatar	94	270	1：2.87	Middel East and North Africa	310 85.16	18–34: 323 88.74% 35–44:14	Mental Illness No 336	NA	NA	118	32.42%	246	67.58%	high mistrust of vaccine safety	9.89%	strong worries about unforeseen	28%	strong concerns about commercial	NA	NA	NA
70	505 teachers vs.745 students from various universities in Bangladesh.	743	507	TeacherL: 1:0.418 Student: 1:0.93	NA	NA	Teachers: 26-65 Students: 16	Corona infected previously :	Completed two doses: Received 1st dose	2: Teacher 462 91.5 Student 455 61.1 1: Teacher: 36 7.1	928	75.90%	312	15.4/32.8	Side effects	(Yes = 1, otherw	long effectivene	0.92/0.86	safety	0.78/0.82	NA	NA
72	students from a local medical school in Anhui, China. Eligible	984	2063	32.3 : 67.7	NA	NA	NA	NA (sELF rated health)?	NA	NA	1154	37.90%	1893	92.10%	Attitudes to the COVID-19 vaccine	How to fill it?荐	Levels of eHealth literacy		Concerns about the COVID-19		NA	NA
73	undergraduate and postgradua	126	543	18.8 : 81.2	Emirati Arab (nation	202 (30.2)	NA	NA	NA	NA	213	31.80%	163	24.40%	Perceived threat and impact of C		COVID-19 vaccination adverse e		Perceived knowledge sufficiency		COVID-19 diagnosis –	Perceived threat and im
74	postgraduate and undergradua	502	398	55.8 : 44.2	NA	NA	NA	NA	NA	NA	648	72.70%	51	5.70%	Anxious about t	30+	Side effect	25+	Inaccurate infor	10+	COVID-19 is not particularly harmful to young	NA
76	students of Taif University	88	244	26.5 : 71.5	NA	NA	18+	NA	NA	NA	278	83.70%	54	16.30%	Safety	Significant deter	Effectiveness	Significant deter	Avoid complica	Significant deter	NA	NA
77	University of Medicine and Ph	215	510	29.6 : 70.4	NA	NA	mean: 22.46	NA	22.75% injected before, dose number not sp		675	93.10%	50	6.90%	Previous COVI	Significant deter	Scientific decisi	Significant deter	Preference for R	Significant deter	NA	NA
79	college students	961	1920	33.4:66.6	NA	NA	19.83+-2.02	NA	NA	NA	2123	76.30%	758	23.70%	Urban/rural	Significant deter	Health related m	Significant deter	Worry to be infe	Significant deter	Worry to be infected	NA
80	undergraduate, graduate, or po	156	958	13.2 : 86.8	NA	NA	30(10.9)	NA	91.6% injected, doses	NA	858	91.60%	76	8.11%	vaccine mandate	Significant deter	views of vaccination as an important	Significant deter	perceptions of a higher risk of	Significant deter	Nearly 20% disagreed that COVID-19 vaccines in Australia	
81	college students	249	406	38.02 : 61.98	NA	NA	18+	NA	NA	NA	413	63.80%	237	36.20%	Trust in the healthcare system	Significant deter	trust in domestic vaccines	Significant deter	NA	NA	NA	NA
82	college students	276	350	44.1:55.9	NA	NA	18+	NA	NA	NA	214	34.20%	412	65.80%	Being third-year	Significant deter	Male	Significant deter	ever watch TV	Significant deter	prefer natural immunity	NA
83	Undergraduate and graduate s	NA			White	66.50%	18+	NA	NA	NA	204	65.60%	54	17.40%	Ethnicity	American Indian or Alaska	Students who received an	Significant deter	a previous COV	Significant deter	NA	NA
85	college students	420	854	32 : 68	Malay	45.80%	17-55	NA	NA	NA	1239	97.25%	35	2.75%	Knowledge of C	Significant deter	risk perception	Significant deter	risk of contractin	Significant deter	risk of contracting	, students' will to get inoculated against COVID-19

Key findings	Confidence (level of trust)				Key findings	Convenience (Access to vaccines, Barriers to				Key findings	Result/Findings	conclusion	Note
	Vaccine		Provider			Assessibility	Availability		Support services				
	Belief in vaccine effectiveness	Side effects	Trust in public health information	Trust in government authorities/healthcare provider		Proximity of vaccination centers, Ease of scheduling	Supply of vaccines	Operational hours	Availability of transportation, Information about vaccination sites		copy the result from the abstract/ main finding	abstract conclusion	
57% of respondents cited concerns about side effects, indicating significant fear related to vaccine safety.	89.4% of participants were either fairly or completely confident that the vaccine was safe.	57% of respondents cited concerns about side effects as a reason for vaccine hesitancy.	13.9% of respondents did not trust public health officials.	41.6% believed the government should not dictate vaccination, indicating trust issues with government	NA	NA	NA	NA	NA	NA	The majority of survey respondents (n = 599) were between the ages of 18–28 (68 %).	Given the worldwide nursing shortage, factors potentially	
Significant concern about severity but not extreme fear of death.		53.5% of overseas and 44.7% of domestic students		Concerns about the government's role were noted but not	Both overseas and domestic university students showed significant intention to vaccinate	NA	NA	NA	NA	NA	A total of 470 overseas university	This study reflected a relatively low	
40% of students were significantly concerned about the severity of COVID-19 but not extreme fear	75% believed in the vaccine's effectiveness.	50% of students were concerned about side effects.	65% trusted public health information.	55% trusted government authorities and		60% of students found vaccination centers easily accessible	58% noted sufficient supply of vaccines.	49% found the operat	NA	NA	The attitudes toward health and safety measures	Our study provides new evidence into	
Students who had a higher fear of COVID-19 (medium to very high fear) were more likely to accept	Trust in the vaccine's safety and effectiveness was a	72.4% of students who refused the vaccine cited doubts	Trust in doctors (71.4%) and government experts	Trust in government was reported by 35.5% of students,	Trust in healthcare providers and pub	NA	NA	NA	NA	NA	Students' answers regarding the COVID-19 and	In the current study, 2249 nursing	
Concerns about side effects were a major reason for hesitancy, with 67.3% citing this factor.	A significant proportion of students who were	67.3% of hesitant students were concerned about side	Vaccine hesitant respondents were less likely to rely on	Trust in healthcare providers and public health information		NA	18.9% of the respondents had received a COVID-	NA	NA	NA	Vaccine hesitancy was prominent in specific racial	This study represents a	
Concern about the adverse effects of the vaccine was a significant issue, with 58.3% of participants	Not directly mentioned, but a majority were willing	58.3% of respondents worried about the adverse	High recommendation of the vaccine by family	Government agencies were a significant source of vaccine-		NA	NA	NA	NA	The study did not provide specific details about the accessibility, availability	Overall, 13,738 (60.6%) of respondents were	More than half of college students were	
74.9% worried about the health of their relatives. 57.2% felt isolated during the pandemic. 34.4% felt	52.8% of the students who wanted to be vaccinated	76.2% preferred to wait until the safety of the vaccines had	31.3% downloaded the contact-tracing mobile app	NA	NA	NA	NA	NA	NA	NA	A total of 1465 healthcare students	Overall vaccine hesitancy among	
34.3% were not worried about the disease and 212 (35.3%) thought that COVID-19 possesses a minor	Among participants, those who agreed that the COVID-19	Concern about side effects was a major reason for vaccine	13.9% did not trust public health officials, contributing	Concerns about government mandates and trust in	Concerns about side effects were significant, with 19.9% fearing side effects. Trust in the effectiveness of	NA	NA	NA	NA	Social media platforms were the main source of COVID-19 vaccine-	We surveyed 600 medical students, 377 (62.8%)	This study revealed low levels of	
59.6% thought they would not be at risk of complications due to COVID-19.	83.0% believed the COVID-19 vaccine is important.	77.4% believed the COVID-19 vaccine might have adverse	77.4% believed vaccine approval guarantees safety.	55.0% thought COVID-19 vaccination should	Generally high confidence in vaccine importance and safety, but significant concerns about side	NA	NA	NA	NA	NA	A total of 1071 of university students were	There is a high rate of acceptability of	
There is a high perception of severity as healthcare students are aware of the significant risk and	High, as 91.7% of respondents accepted the COVID-19	Concerns exist, but specific percentages are not provided.	High, but no data mentioned in the paper	High, contributing to the high acceptance rate of 91.7%.		NA	NA	NA	NA	NA	Out of 384 participants, 91.7% accepted	The current study offers helpful	
Some students (33.3%) did not perceive COVID-19 as severe.	High, as indicated by a 97.8% vaccination acceptance rate.	Concerns about side effects were significant among	High trust in health information is implied by the high	77.8% of hesitant students did not trust public authorities		NA	NA	NA	NA		This study comprised 409 students, most of	Vaccine hesitancy	
NA	7.7% think the vaccines are not effective, 21.9%	Afraid of the side effects (73%)	NA	Do not trust in the government (10.2%)	The spread of negative information on social media about vaccination, such as conspiracy theories,	NA	NA	NA	NA	NA	The acceptance in Vietnam is relatively high	Communication campaigns need to be organized	
Increasing age in the educational system is commonly attributed to higher knowledge and exposure to	NA	NA	NA	Low confidence in politicians, lack of political consensus	The mistrust of the government regarding the COVID-19 vac- cine revealed by the majority of the	NA	NA	NA	NA	NA	The prevalence of COVID-19 vaccine	The prevalence of COVID-19 vaccine	
The perceived susceptibility of university students was lower, which necessities to increase their	50% think the effectiveness of COVID-19 vaccine	Concerned about the side effects (62.6%)	NA	NA	Short-period vaccine development resulted in worries about the side effects of the vaccine, the desire to	NA	NA	NA	NA	NA	In total, over 90% of respondents from	Such findings are very important for	
People worry more about their parents getting the infection especially if they were the source	Belief in the effectiveness and safety of the vaccine	Fear of unknown adverse effects (approximately 22%)	Insufficient information regarding the vaccine	Insufficient trust in the vaccination source	NA	NA	availability of free vaccines (approximately 5%)	NA	NA	NA	Most common deterrent to taking COVID-	Based on our findings, we see that there was a	
actual vaccina- tion was also associated with family experience of infections, and vaccinations	Belief in the effectiveness and safety of the vaccine	117 out 126 respondents who had received vaccination	NA	NA	the actual use of vaccines was less than expected among healthcare providers due to their access to so	NA	availability of free vaccines (11(2.7%))	NA	NA	NA	Of 403 study participants, 50.4% expressed	Half of the study participants	
Perceived severity and general health motivation were not found to be significant predictors.	see high perceived benefits in obtaining the COVID-19	Concerns regarding the safety aspect of vaccination and	Lack of information regarding the effectiveness, safety,	NA	Nursing students had a significantly higher level of concerns about safety and side effects than did medical	Avoid absence from wor	NA	NA	NA	As the salary of medical and nursing students is typically not high, and	The overall intention to receive a COVID-	The most common concerns	This article mainly focus on the comparison
NA	Concerns on safety and quality of COVID-19 vaccines	college student's high concerns on side effects may be	misinformation regarding vaccines and a lack of	"Whether it is recomm	Extant literature suggests that safety concerns and mistrust in vaccines contribute to vaccine hesitancy, and	practice issues (eg. schedule of the vaccine)	NA	Local availability	Provide clear information regarding COVID-19 vaccine access and apply	acceptance group paid more attention on the access to the COVID-19	About 60.6% of the college students reported	Our findings suggest relatively low vaccine acceptance among college students in South Carolina and	
The rapid speed of COVID-19 vaccines' development has raised significant concerns about the	45.2% students are in lack of certainty on the vaccine's		misinformation and conspiracy theories	NA	The rapid speed of COVID-19 vaccines' development, implementation and authorization	NA	NA	NA	NA	NA	In total, 465 students completed the	The current work demonstrated a	
Although the students perceive the importance of the COVID-19 vaccine and agree to make the	Belief in the effectiveness and safety of the vaccine	Fear of unknown adverse effects (56.31%)	NA	Insufficient trust in the vaccination source (producer).	Hesitancy is exacerbated by social media, conspiracy theories, and misinformation. Evidence suggests	NA	NA	NA	NA	NA	The majority of the participant students (90.5%)	The COVID-19 vaccine acceptance was	
more than 50% of health profes- sions students agreed on beliefs about the susceptibility to COVID	Vaccine effectively prevents the spread of the SARS-CoV-2	Manufacturers do not disclose information about	Mass media appreciate the effectiveness and	Health authorities ensure safe and effective vaccination	60% of health professions students agreed on confirma- tion of the effectiveness and safety of the	Difficult access to vaccination if not free (40.3%)	NA	NA	NA	NA	A total of 911 heath professions students	Health professions students'	
NA	Belief in benefits of vaccine, perceived effectiveness of	fear of side effects	influence of social environment	Trust in instituitions		accessibility, time	NA	NA	NA	NA	Data from 872 eligible respondents were	This study highlights which aspects	
An individual's perception of the infection risk is directly proportional to the degree of	NA	Be confident in vaccine safety (87.3%); No adverse	NA	Government encouragement and sound policies	Lack of knowledge has been reported as the most critical reason for vaccine hesitancy. People with	NA	Worry about vaccine	NA	Convenient channels to vaccination (85.6%); inconvenient channels	NA	Among the 721 participants (aged 18 to 23 years)	This study reflected Chinese	
The finding is supported by a meta analysis entitled 'anticipated regret and health behaviour states that	5 out of 247 believe in the effectiveness and safety of the	fear of side effects, 39%, concerned about its safety,	NA	NA	The possible justification might be due to the vaccine development period were not taken the adequate	availability of free vaccines	NA	NA	NA	NA	The present study showed that the odds of COVID-	COVID-19 vaccine hesitancy is found to be high. Therefore, students are advised to receive COVID-19 vaccine [illegible] of vaccines and their benefits, they will be able to have a greater impact on the rest of the	
The percentage of vaccinated university students (66.7%) was relatively lower compared to a	30.1% of participants have concerns over the efficacy of the	58.7% of participants have concerns about the side effects of	74.6% of participants in Saudi agreed that social media carries	57.1% of participants agree with the belief of complete		NA	NA	NA	NA	NA	A total of 385 par ticipants consented to the	COVID-19 vaccination rates among	
Risk perception of self-regarding COVID-19 increased the students' willingness to participate in	28.3% of participants have concerns over efficacy of vaccine	82.3% of students have concerns about safety of vaccine	74.8% of participants trust the information they are receiving	74.8% of participants trust the information they are receiving		NA	NA	NA	NA	NA	Vaccine hesitancy was found among 10.6%. Concern	COVID-19 vaccine hesitancy was	
	29.3% of participants have concerns about the efficacy of	The scoring rate of people who believe "I am afraid of the	31% of participants claim their lack of information about	1.7% of participants worry about government		152 (38.3%) participants said there are 0-2 medical units that can	NA	NA	NA		A total of 397 questionnaires were analysed,	In general, most of the participants had	
ight spread COVID-19 if I get infe	The odds ratio for "I I	The odds ratio for "I a	Less accessibility of p	Mant et al. [25] found that 80% of students who were unwi		NA	NA	NA	NA		Results showed that 56.7% of students had	The public health strategy to improve	
	34 participants believ	35 participants believ	401 participants belie	58 participants believe that government is using the vaccin		NA	NA	NA	NA		Overall, participants were vaccine accepting	The widespread prevalence of SARS-CoV-2	
	54.1% of respondents	43.5% of respondents	65.6% of respondents	65.5% of respondents trust the public health information fr		NA	NA	NA	NA		Nearly, all participants had positive attitudes	Our results highlight the need for an	The article mentions that the survey was
	8.7% of respondents	79.3% of respondents	NA	11.9% of respondents do not trust the government as one r		NA	NA	NA	NA		A total of 412 eligible participants were	The theoretical framework recognized	
gree that "Because of the low sprea	68.7% of respondents	42.2% of respondents	NA	NA		73.6% of respondents in	NA	NA	74.4% of respondents indicate that "I have the ability to obtain information		In this cross-sectional study, an online	In conclusion, the level of acceptance of	
gree that "It is necessary for me to	13.6% of respondents	32.7% of respondents	NA	NA		55% of respondents agre	NA	NA	NA		41.2% vaccine hesitancy was reported among	Confidence in vaccines and perceptions of	
The result indicates people's fear of COVID-19	Most participants believe vaccine is effective (score	Most participants think that the vaccines available on			The result indicates people's trust of vaccine	NA	NA	NA	NA		A total of 1488 valid questionnaires	Nursing students showed	No exact data for number of patients, so use
The results indicates that many participates do not think they need vaccine to prevent COVID-19	36.5% participants are not sure of its effectiveness.	39.6% participants are concerned of the vaccine safety. 21.9	35.4% participants have heard or read negative information	13.5 % participants believe vaccine development was	The results indicate that participants are concerned about its side effects the most, then its effectiveness and	NA	5.2% participants think the vaccine brand they prefer	9.4% participants are busy with studies (no time)	4.2% participants don't know where to get vaccinated	The results indicates that opetional hours take the largest portion in this	Out of the 281 students who received the	This study has revealed a high level of	
The result indicates that many participants do not think the risk of infection is high enough to get	8.4% participants concerns about the ineffectiveness of	58.64% participants concerns about the ineffectiveness of	3.49% participants are concerned about absence of confirmed	40.89% participants do not comply with health ministry	On this side, concerns about side effects take the largest portion, while the concerns about lack of vaccined	NA	NA	NA	NA		Overall, 491 (77.81%) students actually	Our study showed higher COVID-19	
	Fear of ineligibility (39.32%);	Fear of side effects (87.18%)	Lack of information (70.94%)	NA	The results indicate that participants are concerned about its side effects the most, then the lack of	NA	Probable cost of vaccination (5.98%)	NA	NA	The results indicates that inconvenience is not the main reason for vaccine	Overall, 58.13% and 64.81% of university	The findings from this study can aid	
Participants accept vaccine to protect the community more than themselves	NA	52% participants believe no one get sick from vaccine;	NA	43.1% participants believe the recommendation of	Most participants trust the vaccine is safe (no or very little side effcts)	NA	NA	NA	NA		Medical students scored higher for prior knowledge	The majority of non-medical students	
	42.5% participants believe the durantion of protection	83.6% participants believe their knowledge of	NA	34.6% participants believe advice of their doctor can	The concern of side effects takes the largest portion, followed by the trust of effectiveness in the level of trus	NA	NA	NA	NA		Overall, 26.9% of participants reported being	Our findings help guide future efforts to	
Participants feel hesitant about vaccine mainly due to their low perception of individual risk	48.7% participants voting for acceptavtans believe	34% participants refusing vaccines are concerned about side	NA	NA	Participants are concerned about the effectiveness and side effect of vaccine	NA	NA	NA	NA		Most participants (72.4%) reported having adequate	Ajman dental students had moderate knowledge of COVID-19 and obtained	
Participants worry about risk from others more.	63.3% participants thin being vaccinated is a great idea,	36.7% participants worry that their normal life will be	NA	59.7% of them concerned about the authenticity of the	Participants hold a strong belif in the effectiveness of vaccine when it comes to acceptance; on hesitation,	NA	39.7% of them are concerned about the affordability of the	NA	NA	Some participants worry about the affordability of vaccine	We received 330 responses. About 36.4% intended	The correct and comprehensive beliefs of the	
	NA	7.5% participants (n=813) are concerned about side	NA	2.83% participants lack trust of government	Participants are most concerned about side effect of vaccine	NA	NA	NA	NA		After adjusting for social demographics	Public health messages should utilize	
	35.1% (n=164) have general mistrust of the benefits of	35.8% reported that side-effects will prevent them from	NA	66% perceived that the government would make the		NA	NA	NA	NA		Encouragingly as well, 66% of the participants	the participants had a good knowledge	
	630 (58.9%) of the participants have confidence in	396 (37%) percent of the participants agree that COVID-	Socialmedia is a reliable source forCOVID-19	NA		NA	NA	NA	NA		Of the 1069 subjects, 629(58.8%) were	Having had a flu vaccine in the past, social	
COVID-19 vaccine refusal concerns were perceived less in dividual COVID-19 risk, the	The main reasons (perceived barriers) for participants'	had concern on side effects 68%	NA	NA	In this re gard, the concerned stakeholders such as the federal ministry of health, the regional health	NA	NA	NA	NA	NA	The proportion of the COVID-19 vaccine	Though, this study found a 69.3%	
NA	25 (10.2%) of the patients that showed hesitancy believed	Out of the 246 participants who were hesitant to		71.1% felt that the COVID-19 vaccines did not pass through	Vaccine safety and effectiveness were the main reasons for This includes concerns that the COVID-	NA	NA	NA	NA	NA	Of the 326 participants, 98.8% were	There was significant vaccine	In our study, those who were married and not
NA	NA	NA		Confidence in the experts of the Civil Protection	Additionally, students of health sciences display greater adherence to epidemiological measures and have	NA	NA	NA	NA	NA	Results show that the majority of students of	Therefore, improving health literacy	
NA	Confidence (the level of trust in the effectiveness) with	NA	General media trust: association: insignificant	Topic-specifc trust in news sources: Federal government:	Confdence in the safety of vaccinations is crucial for vaccinatio uptake. Tus, for a COVID-19	NA	NA	NA	NA	NA	Psychological factors showed high	Our fndings can help better	
Our study depicted that based on the adequate information provided to the students, it boosts	the majority of the study participants, about	The finding reveals that about 37.8% agreed that	NA	NA	NA	NA	NA	NA	NA	NA	The data statistics show that 4.9% of the	COVID-19 vaccination acceptability	
NA	Perceived benefits 20.48 ±2.61 19.75±2.55	Previous studies indicated that side effects,	NA	NA	NA	NA	Perceived barriers Yes:14.53 ±2.58 No:15.97±2.24 OR:	NA	NA	Participants who perceived high barriers to vaccination	: Out of 384 participants, 91.7% accepted	The current study offers helpful	
NA	Reason for not taking COVID-19 vaccine vaccine not effective	Fear of side effect 68 16.9	NA	NA	NA	NA	NA	NA	NA	NA	e. The prevalence of COVID-19 vaccine uptake	In conclusion, majority of the respondents	
NA	NA	Fear of potential serious side effects 45 (61.6%)	Lack of trust in vaccine manufacturers/public	NA	isk of contracting the disease, perceived low efficacy of the vaccine, fear of side effects, and low	NA	NA	NA	NA	NA	Overall, 214 medical students participated in the	The study found a high vaccine uptake	
A quarter of the participants did not agree that receiving the vaccine would make them less	I am concerned about the efficacy of the COVID-19	I am worried that the possible side-effects of COVID-19	NA	NA	NA	NA	The availability of the vaccine had an impact on n =	NA	NA	NA	There were 715 participants and 556 who	Vaccines are integral in the prevention of	
Men were 48% less likely to reach the threshold value on the CAS Scale compared to women	Of the 364 participants, 9.89% expressed a	28% expressed strong worries about unforeseen side	NA	NA	NA	NA	NA	NA	NA	NA	Of the 364 participants, 9.89% expressed		. Our findings differ from the data on vaccine
NA	Booster shot has long effectiveness to protect COVID-19	Booster doses will produce severe side effects (Yes = 1,	I trust that booster doses provides long-term herd immunity	NA	NA	NA	NA	NA	NA	NA	The pooled COVID-19 vaccine booster	COVID-19 booster vaccine acceptability	
NA	Difference	NA	NA	NA	NA	Place of residence no sig	NA	NA	NA	NA	main reasons for the low acceptance rate		
NA	NA	NA	NA	NA	NA	NA	NA	NA	NA	NA	The main factors that explained vaccine hesitancy		
NA	NA	NA	NA	NA	NA	NA	NA	NA	NA	NA	The results unearthed that female		
	Significant	Significant	NA	Physician recommendation		NA	NA	NA	NA	NA	Out of 332 participants, 278 (83.7%)		
	NA	NA	NA	NA		NA	NA	NA	NA		3.1% of students presented a strong intention		
	Keep you free of CO	Feel vaccine safe	NA	NA		NA	NA	NA	NA		Totally, 2,881 college students participated in		
		Nearly 20% disagreed that COVID-19		Participants consider COVID-19 informa_x0002_tion from		NA	NA	NA	NA		Most participants were enrolled in nursing programs		
	trust in domestic vaccines	NA	NA	Trust in the healthcare system		NA	NA	NA	NA		27.8% of the students indicated that they had		
	Not effective for CO	Fear of side effect 59.4%			Lack of detailed info about the vacci	NA	NA	NA	NA		In this study, the student's willingness to		
	NA	NA	NA	NA		NA	NA	NA	NA		Results: The results of this		
	NA	risk perception	NA	did not influence		NA	NA	NA	NA		the majority of university students had		
